# Construction of ferroptosis-related prediction model for pathogenesis, diagnosis and treatment of ruptured abdominal aortic aneurysm

**DOI:** 10.1097/MD.0000000000038134

**Published:** 2024-05-10

**Authors:** Ailu Wang, Li Zhou

**Affiliations:** a Department of Neonatology, the First Hospital of China Medical University, Shenyang, China; b Department of Geratology, the First Hospital of China Medical University, Shenyang, China.

**Keywords:** diagnosis, ferroptosis-related prediction model, ruptured abdominal aortic aneurysm, targeted inhibition

## Abstract

Abdominal aortic aneurysm (AAA) is a dangerous cardiovascular disease, which often brings great psychological burden and economic pressure to patients. If AAA rupture occurs, it is a serious threat to patients’ lives. Therefore, it is of clinical value to actively explore the pathogenesis of ruptured AAA and prevent its occurrence. Ferroptosis is a new type of cell death dependent on lipid peroxidation, which plays an important role in many cardiovascular diseases. In this study, we used online data and analysis of ferroptosis-related genes to uncover the formation of ruptured AAA and potential therapeutic targets. We obtained ferroptosis-related differentially expressed genes (Fe-DEGs) from GSE98278 dataset and 259 known ferroptosis-related genes from FerrDb website. Enrichment analysis of differentially expressed genes (DEGs) was performed by gene ontology (GO) and Kyoto encyclopedia of genes and genomes (KEGG). Receiver Operating characteristic (ROC) curve was employed to evaluate the diagnostic abilities of Fe-DEGs. Transcription factors and miRNAs of Fe-DEGs were identified through PASTAA and miRDB, miRWalk, TargetScan respectively. Single-sample gene set enrichment analysis (ssGSEA) was used to observe immune infiltration between the stable group and the rupture group. DGIdb database was performed to find potential targeted drugs of DEGs. GO and KEGG enrichment analysis found that DEGs mainly enriched in “cellular divalent inorganic cation homeostasis,” “cellular zinc ion homeostasis,” “divalent inorganic cation homeostasis,” “Mineral absorption,” “Cytokine − cytokine receptor interaction,” “Coronavirus disease – COVID-19.” Two up-regulated Fe-DEGs MT1G and DDIT4 were found to further analysis. Both single and combined applications of MT1G and DDIT4 showed good diagnostic efficacy (AUC **= **0.8254, 0.8548, 0.8577, respectively). Transcription factors STAT1 and PU1 of MT1G and ARNT and MAX of DDIT4 were identified. Meanwhile, has_miR-548p-MT1G pairs, has_miR-53-3p/has_miR-181b-5p/ has_miR-664a-3p-DDIT4 pairs were found. B cells, NK cells, Th2 cells were high expression in the rupture group compared with the stable group, while DCs, Th1 cells were low expression in the rupture group. Targeted drugs against immunity, GEMCITABINE and INDOMETHACIN were discovered. We preliminarily explored the clinical significance of Fe-DEGs MT1G and DDIT4 in the diagnosis of ruptured AAA, and proposed possible upstream regulatory transcription factors and miRNAs. In addition, we also analyzed the immune infiltration of stable and rupture groups, and found possible targeted drugs for immunotherapy.

**Key Points**

1. We explored the potential mechanisms of the occurrence and development of ruptured abdominal aortic aneurysm (AAA) based on ferroptosis-related prediction model.
2. We found the clinical significance of ferroptosis-related differentially expressed genes (Fe-DEGs) MT1G and DDIT4 in the diagnosis of ruptured AAA.
3. Multiple upstream regulatory transcription factors and miRNAs of MT1G and DDIT4 were observed.
4. We predicted GEMCITABINE-FKBP5 and INDOMETHACIN-ADM as possible targeted drugs to inhibit MT1G and DDIT4.

## 1. Introduction

Abdominal aortic aneurysm (AAA) is a degenerative cardiovascular disease that occurs most frequently in men over 65 years old and increases with age.^[1,2]^ The most dangerous complication of AAA is rupture, which has a mortality rate of up to about 70%.^[3,4]^ At this point, open surgery and intracavitary repair are inevitable choices, but for patients with poor basic conditions, intracavitary repair can only be selected to minimize the additional trauma caused by treatment.^[5]^ However, the mortality and complications associated with both therapies remain significant.^[6]^ Therefore, it is urgent to explore the pathogenesis of AAA, improve existing treatment measures, and even develop new therapeutic methods.

AAAs are characterized by local weakness and irreversible dilation. Oxidative stress and apoptosis of vascular smooth muscle cells, the main component of artery middle membrane, play important roles in the occurrence and development of AAA.^[7,8]^ Nonapoptotic forms of cell death may also be activated under specific pathological conditions. In 2012, the concept of ferroptosis was first proposed by Scott J to describe a specific cell death process due to unusual biochemical and physical properties.^[9]^ Specifically, Ferroptosis is an iron-dependent regulated cell death that occurs when lipid peroxidation accumulates.^[10,11]^ It has been shown that the formation of AAA is locally accompanied by a large number of oxidative stress products.^[7,12]^ Therefore, we consider whether ferroptosis is involved in the pathogenesis of AAA and whether it affects aneurysm rupture.

Based on the above hypothesis, we downloaded stable and ruptured AAA data from GEO database, and ferroptosis-related genes (FRGs) from FerrDb website. Through the combination of the 2, the possible pathogenesis of ruptured AAA is analyzed.

## 2. Materials and methods

### 2.1. Data extraction

Gene expression profiles of AAA patients were obtained from GEO database (https://www.ncbi.nlm.nih.gov/geo/). GSE98278, conducted by GPL10558(Illumina HumanHT-12 V4.0 expression beadchip), included mRNA data of 31 stable AAA samples and 17 ruptured AAA samples. We acquired 259 FRGs from FerrDb website (http://www.zhounan.org/ferrdb/), which included markers, regulators, and inducers of ferroptosis (see Table S1, Supplemental Digital Content, http://links.lww.com/MD/M474, which lists 259 FRGs from FerrDb website which included markers, regulators, and inducers of ferroptosis).

### 2.2. Differential expression analysis

The differentially expressed genes (DEGs) were analyzed between stable and rupture groups by performing the limma R package in R studio (version 4.1.1) with the statistical threshold of |logFC| > 1 and false discovery rate (FDR) < 0.05.

### 2.3. Gene functional enrichment analysis

Gene ontology (GO) and Kyoto encyclopedia of genes and genomes (KEGG) pathway enrichment analysis were performed for these DEGs by using the “cluster Profiler” R package with *P* < .05 and FDR < 1.

### 2.4. Ferroptosis-related DEGs analysis

Calculate and draw custom Venn diagrams (http://bioinformatics.psb.ugent.be/webtools/Venn/) was used to identified ferroptosis-related DEGs (Fe-DEGs).

### 2.5. Diagnostic value analysis

Receiver operating characteristic (ROC) curves were drawn and the area under the ROC curve (AUC) was calculated for these Fe-DEGs by using GraphPad Prism (V.8.0).

### 2.6. Transcription factors analysis

PASTAA and TRAP (http://trap.molgen.mpg.de/) were performed to predict transcription factors that regulated Fe-DEGs.^[13,14]^ JASPAR (https://jaspar.genereg.net/) was used to visualize binding sites of transcription factors to promoter regions of Fe-DEGs.^[15]^

### 2.7. miRNA analysis

miRNAs regulating Fe-DEGs were obtained through miRDB (http://mirdb.org/), miRWalk (http://mirwalk.umm.uni-heidelberg.de/), and TargetScan (http://www.targetscan.org/) databases. TargetScan was implemented to show the overlapping miRNA among these 3 databases binding with 3’ – untranslated regions of Fe-DEGs.

### 2.8. Immune infiltration analysis

ESTIMATE was used to evaluate the immune cell infiltration level (immune score), matrix content (stromal score) and comprehensive score (ESTIMATE score) between stable and rupture groups.^[16]^ The “single-sample gene set enrichment analysis (ssGSEA)” was performed to analyzed infiltrating score of 16 immune cells and activity of 13 immune-related functions between stable group and rupture group.^[17]^

### 2.9. Drug analysis

DGIdb (https://dgidb.genome.wustl.edu/) is an open-source project that offers user-friendly browsing, searching, and filtering of information on drug-gene interactions and the druggable genome.^[18]^ The parameters were set as: FDA approved; Immunotherapies. The protein data bank (PDB) (https://www.rcsb.org/) contains 3D shapes of proteins, nucleic acids and complex components to help students and researchers understand all aspects of biomedicine and agriculture, from protein synthesis to health and disease.^[19]^ PharmMapper (http://www.lilab-ecust.cn/pharmmapper/) is a freely accessed web server designed to identify potential target candidates for the given probe small molecules (drugs, natural products, or other newly discovered compounds with binding targets unidentified) using pharmacophore mapping approach.^[20]^

### 2.10. Statistical analysis

Mann–Whitney *U* test was conducted to compare data between different groups. Statistical analysis and data visualization were performed by using R Studio (V.4.1.1), GraphPad Prism (V.8.0) and AutoDock (V.4.2) software. *P* < .05 was considered statistically significant.

## 3. Results

### 3.1. Identification of DEGs

**Figure 1.**
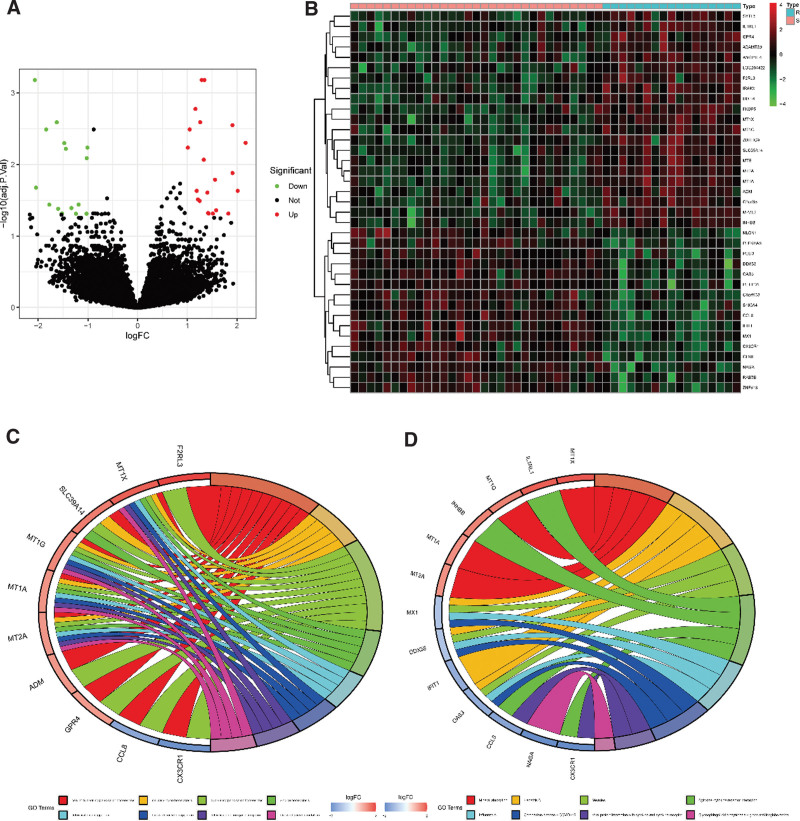
(A–B) The volcano map (A) and the Heatmap (B) of differentially expressed genes (DEGs) in 2 groups. (C–D). The GO (C) and KEGG (D) analysis of DEGs in different groups. GO = gene ontology, KEGG = Kyoto encyclopedia of genes and genomes.

DEGs were analyzed between the stable group and the rupture group from GSE98278, and the results as shown in the volcano map (Fig. 1A) and the Heatmap (Fig. 1B), contained 21 up-regulated genes and 16 down-regulated genes (see Table S2, Supplemental Digital Content, http://links.lww.com/MD/M475, which lists 21 up-regulated genes and 16 down-regulated genes of DEGs results analyzed between the stable group and the rupture group from GSE98278). GO and KEGG analysis found that these genes mainly enriched in “cellular divalent inorganic cation homeostasis,” “cellular zinc ion homeostasis,” “divalent inorganic cation homeostasis,” “Mineral absorption,” “Cytokine–cytokine receptor interaction,” “Coronavirus disease – COVID-19” (Fig. 1C–D).

### 3.2. Diagnostic value of Fe-DEGs

**Figure 2.**
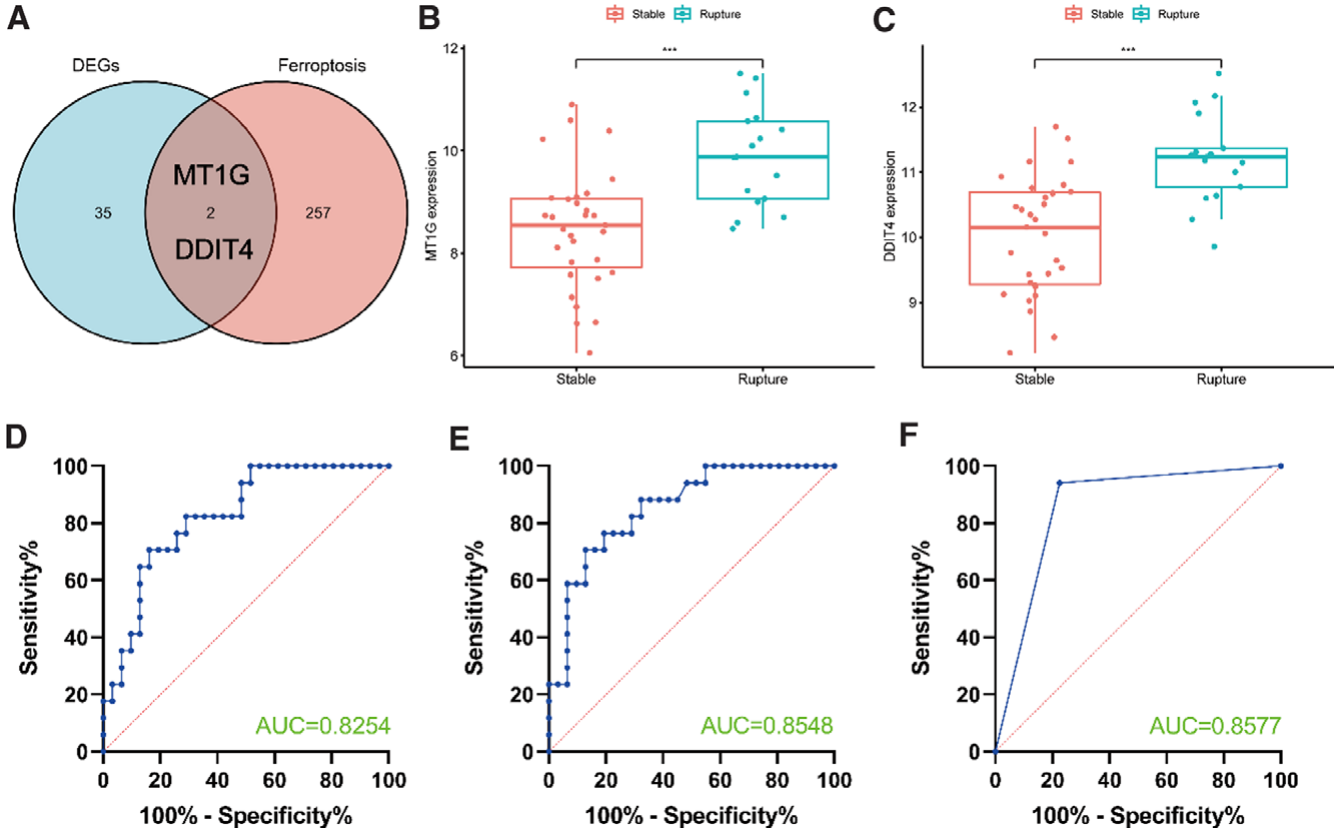
(A) The Venn diagram showed the intersection of ferroptosis-related genes and DEGs. (B–C) The expression levels of MT1G and DDIT4 in stable and rupture group. (D–F) The results of ROC curve for MT1G, DDIT4 and “MT1G + DDIT4.” DEGs = differentially expressed genes, MTs = metallothiosin, ROC = receiver operating characteristic.

The Venn diagram showed the intersection of FRGs and DEGs were MT1G gene and DDIT4 gene (Fig. 2A). Meanwhile, we found that MT1G and DDIT4 were higher expression in the rupture group compared to the stable group (Fig. 2B and C). Then, we explored their diagnostic valve, and the results revealed that AUC based on MT1G and DDIT4 were 0.8254 and 0.8548, respectively (Fig. 2D and E), which represents a good performance. Besides, we further analyzed the combined diagnostic capability of the MT1G and DDIT4, which also had a nice result (AUC = 0.8577) (Fig. 2F).

### 3.3. Mechanism of ruptured AAA

**Figure 3.**
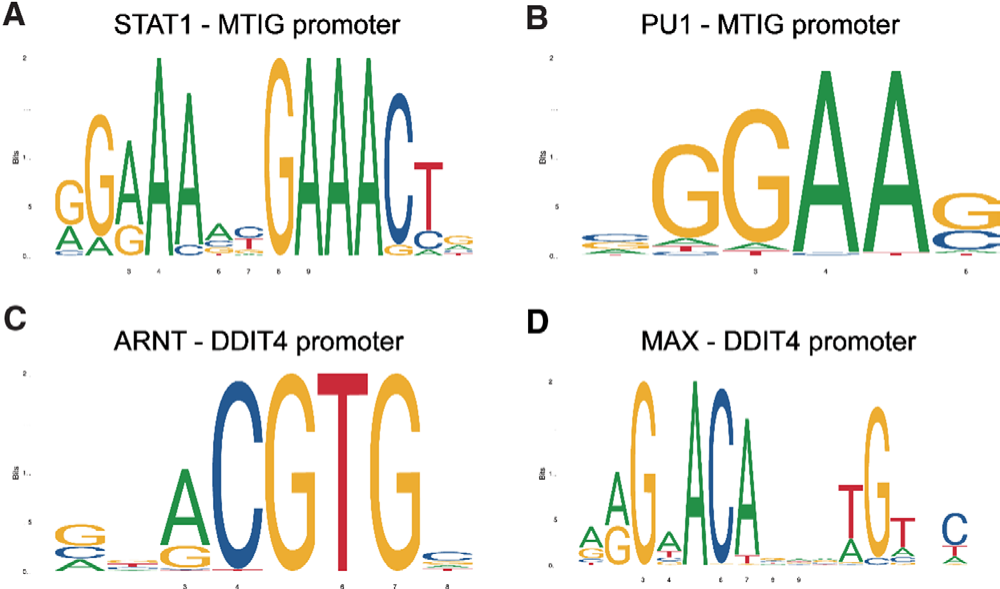
(A–D) The possible specific binding sites of MT1G and DDIT4. MTs = metallothiosin.

Given that they were highly expressed in the rupture group, we predicted regulatory transcription factors of MT1G and DDIT4. The top 5 are listed in Table 1. And specific binding sites of the top 2 (STAT1 and PU1, ARNT, and MAX) were shown in Figure 3.

**Table 1:** The top 5 regulatory transcription factors of MT1G and DDIT4.

Rank	*P* value	Corrected_*P* value	Matrix_ID	Matrix_name
	MT1G			
1	.001921236	.841163638	M01212	STAT1_Q3
2	.005618119	.841163638	M01203	PU1_01
3	.006611339	.841163638	M00929	MYOD_Q6_01
4	.007230223	.841163638	M00054	NFKAPPAB_01
5	.027361843	.841163638	M00648	MAF_Q6
	DDIT4			
1	.017711669	.985959635	M00539	ARNT_02
2	.020521541	.985959635	M00119	MAX_01
3	.029893506	.985959635	M00927	AP4_Q6_01
4	.034127139	.985959635	M00054	NFKAPPAB_01
5	.037773204	.985959635	M01288	NEUROD_02

**Figure 4.**
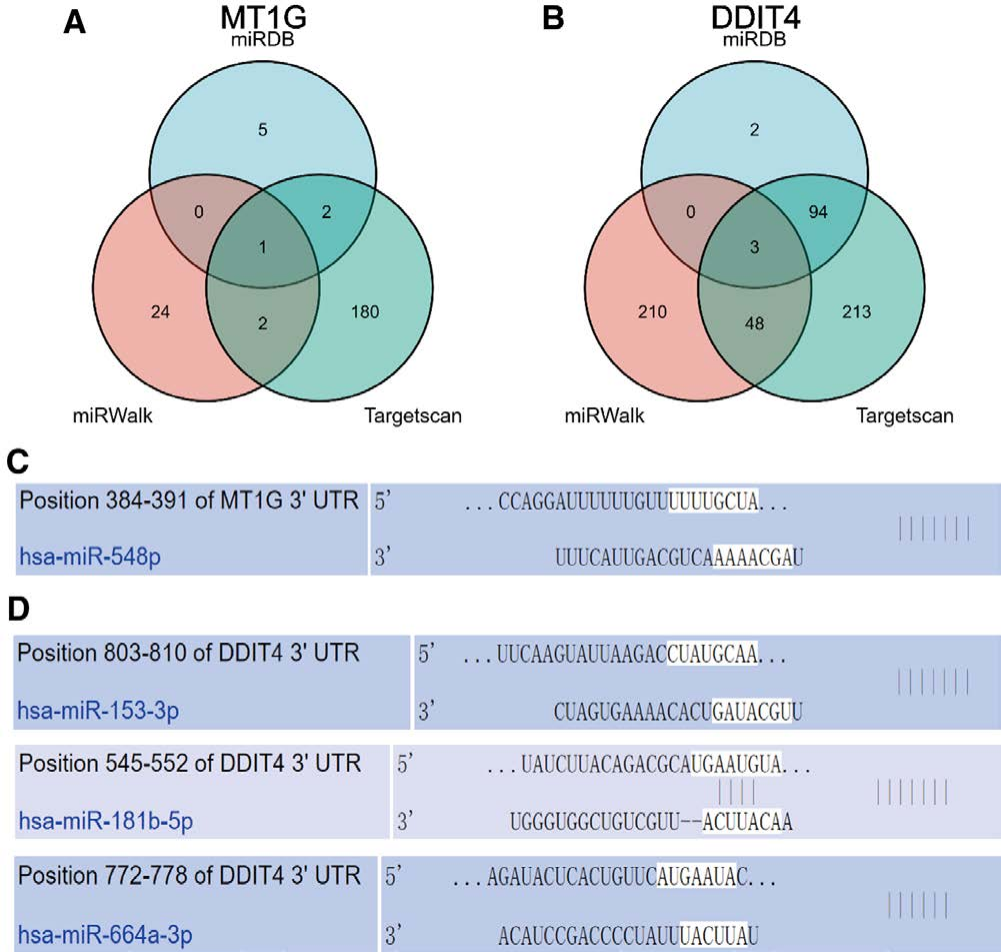
(A–B) These common miRNAs of MT1G (A) and DDIT4 (B) in 3 databases. (C–D) The specific binding sites of common miRNAs and MT1G (C) and DDIT4 (D). MTs = metallothiosin.

On the other hand, we utilized miRDB, miRWalk and TargetScan databases to predict upstream regulatory miRNAs of MT1G and DDIT4 (see Table S3, Supplemental Digital Content, http://links.lww.com/MD/M476, which lists prediction of upstream regulatory miRNAs of MT1G and DDIT4). To increase the reliability of prediction, we identified common miRNAs in 3 databases for subsequent analysis, containing has_miR-548p, has_miR-53-3p, has_miR-181b-5p, has_miR-664a-3p (Fig. 4A and B). Finally, we downloaded images of specific binding sites from TargetScan databases (Fig. 4C and D).

### 3.4. Relationship between immune infiltration and ruptured AAA

**Figure 5.**
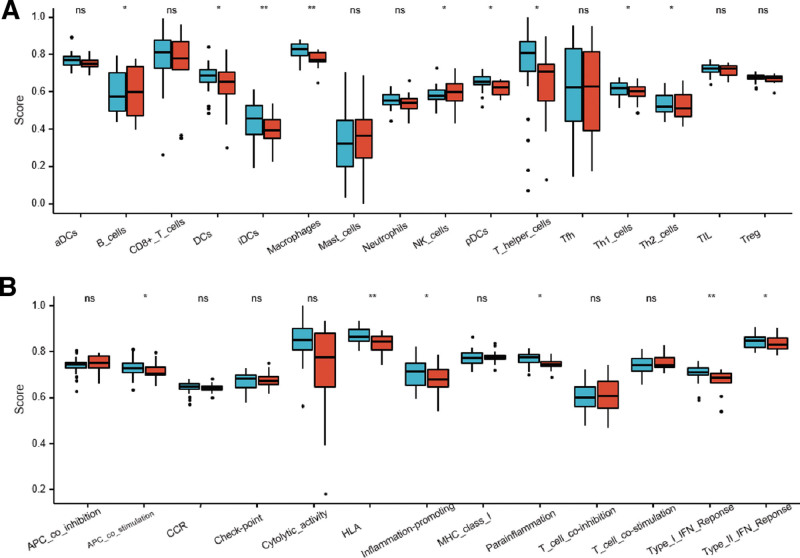
The immune cells and immune function of ssGSEA between the stable group and the rupture group. ssGSEA = single-sample gene set enrichment analysis.

To investigate the association between immune infiltration and ruptured AAA, we analyzed the differences in immune cells and immune function between the stable group and the rupture group by ssGSEA. The results manifested that B cells, NK cells, Th2 cells were significantly high expression in the rupture group compared with the stable group, while DCs, iDCs, pDCs, Th1 cells, APC_co_stimulation, HLA, inflammation-promoting, parainflamation, Type_I_IFN_Reponse, Type_II_IFN_Reponse were significantly low expression in the rupture group (Fig. 5A and B).

### 3.5. Exploration of targeted drug

**Figure 6.**
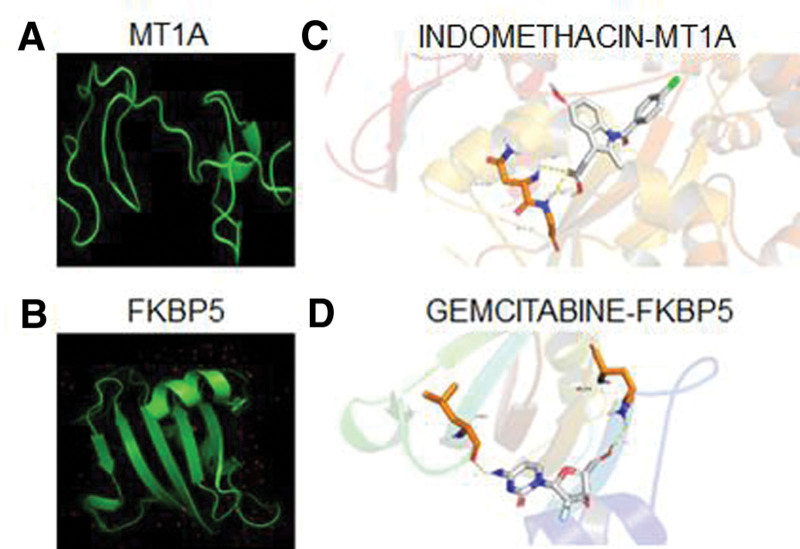
Potential drug target prediction for MT1A and FKBP5. (A–B) Three-dimensional structure of MT1A and FKBP5. (C–D) Molecular docking sites of INDOMETHACIN-MT1A and GEMCITABINE-FKBP5. MTs = metallothiosin.

We input the up-regulated DEGs into the DGIdb database, and the parameters were set as Approved and Immunotherapies based on ruptured AAA closely related to immune infiltration. Surprisingly, we found 3 medications may be effective to control AAA rupture. Subsequently, we found the tertiary structure of drugs (INDOMETHACIN, GEMCITABINE) and target genes (MT1A, FKBP5) through PharMmapper and PDB database (Fig. 6A and C), and then evaluated the docking among them by AutoDock software. The results showed that INDOMETHACIN-MT1A and GEMCITABINE-FKBP5 had a relatively strong binding ability (Fig. 6B and D).

## 4. Discussion

Rupture of AAA is a serious complication, which brings great health threat and economic burden to patients. Oxidative stress and apoptosis of vascular smooth muscle cells are 2 clear pathologic forms of AAA. Recent studies have shown that almost all recognized ferroptosis regulators regulate cell redox and cell cycle,^[21,22]^ which happens to coincide with the pathogenesis of AAA. Therefore, by analyzing large sample data and combining it with the new form of cell death, our study explored the pathogenesis and potential drug targets of ruptured AAA.

First, we obtained microarray data of stable and ruptured AAA samples from the GEO database. Then we identified DEGs related to ferroptosis between the 2 groups, and 2 up-regulated genes MT1G and DDIT4 were selected. Metallothiosin (MTs) is a family of low molecular weight cysteine-rich intracellular proteins that play a key role in antioxidants and homeostasis of biologically essential metals, such as zinc, copper, and iron.^[23,24]^ MT1G is an important subtype of MTs and views as a tumor suppressor in different types of cancer. Studies have reported that MT1G can upregulate the expression of P21 and Bax by interacting with p53, leading to cell cycle arrest and apoptosis of HCC cells, respectively.^[25]^ It has also been reported that MT1G can inhibit the proliferation and invasion of thyroid cancer and induce apoptosis.^[26]^ Unfortunately, there are few reports about MT1G in cardiovascular diseases, and this study can make up for this. DNA damaging-induced transcript 4 (DDIT4) is induced by various cellular stress conditions, such as hypoxia, endoplasmic reticulum stress and oxidative stress.^[27]^ DDIT4 can inhibit the activity of rapamycin complex 1 (mTORC1), which is a major participant in cell growth, proliferation and survival, and thus regulate cell apoptosis.^[28]^ Importantly, DDIT4 has been shown to promote apoptosis and autophagy in myocardial infarction,^[29]^ insulin reperfusion,^[30]^ ataxia telangiectasia^.[31]^ This provides a greater possibility for DDIT4 to participate in the evolution of ruptured AAA.

Having identified the functions of MT1G and DDIT4, we wanted to understand their potential as biological markers. The results showed that MT1G alone and DDIT4 alone had 0.8254 and 0.8548 diagnostic efficacy in predicting AAA rupture, respectively. The combined ability of MT1G and DDIT4 was similar to that of the 2 alone. However, the objective problem is that the data contained in the online database are artery wall samples rather than serum or blood cell samples. Whether the 2 can be promoted as clinical diagnostic indicators to distinguish AAA rupture remains to be further verified. Nevertheless, MT1G and DDIT4 give rise to further thinking about the pathogenesis of ruptured aneurysms.

In order to explore the upstream regulatory factors of MT1G and DDIT4, on the 1 hand, we found specific binding sites between multiple transcription factors and our target genes through PASTAA database, and the JASPAR database verified their specific binding sites again. On the other hand, we used 3 databases to jointly predict upstream regulatory miRNAs of MT1G and DDIT4. Has_miR-548p and MT1G, has_miR-53-3p, has_miR-181b-5p, has_miR-664a-3p and DDIT4 were successfully screened out. And the TargetScan database is executed to publish specific regulatory sites. Has_miR-548p has been reported to interact with the 3’-untranslated region of apoB mRNA to enhance posttranscriptional degradation to reduce lipoprotein production and lipid synthesis.^[32]^ Has_miR-181b-5p can enhance cell proliferation and reduce apoptosis by regulating the MEK/ERK/ P21 pathway.^[33]^ The roles of has_miR-53-3p and has_miR-664a-3p are reported for the first time in this paper. According to the clear pathogenesis of AAA, hyperlipidemia is a risk factor for AAA, and apoptosis of vascular smooth muscle cells is a key pathological change of AAA. According to the reported functions of has_miR-53-3p and has_miR-664a-3p, they have a good potential to inhibit the formation and progression of AAA. However, although we found the upstream regulatory mechanism of MT1G and DDIT4, these results lack further verification and clarification of biological experiments, and the reliability of the results can be improved by joint prediction of multiple databases.

Next, we further explored the differences of immune infiltration between stable and rupture groups, and found that B cells, NK cells, and Th2 cells were significantly increased in the rupture group. The disequilibrium of immune cell distribution between the 2 groups has also been reported by other studies.^[34]^ Therefore, when we screen targeted drugs, we focus on proven immunotherapy-related drugs, which also improves the possibility of transformation to some extent. In addition, immune regulation as a treatment method for AAA is also recognized and promising.^[35]^ Ultimately, we found 2 drugs that might prevent AAA rupture, including GEMCITABINE, and INDOMETHACIN. GEMCITABINE is classic chemotherapy drug for tumors, but it is rarely used in chronic diseases. We believe that with continuous research, this drug may also become new treatments to inhibit the progression of AAA. About the INDOMETHACIN, Vascular Surgical Society of Great Britain and Ireland as early as 1999 proposed the strategy that nonsteroidal anti-inflammatory drugs treat abdominal pain.^[36]^ Subsequent clinical studies found that INDOMETHACIN could limit the expansion of small AAA.^[37]^ Recent animal experiments have shown that INDOMETHACIN reduces the incidence of abdominal aortic rupture by inhibiting monocyte/macrophage accumulation in mouse models.^[38]^ Of course, the promotion of INDOMETHACIN in the treatment of AAA requires data from a large multi-center sample.

## Author contributions

**Conceptualization:** Ailu Wang.

**Data curation:** Ailu Wang.

**Formal analysis:** Ailu Wang.

**Investigation:** Ailu Wang.

**Methodology:** Ailu Wang, Li Zhou.

**Project administration:** Li Zhou.

**Resources:** Li Zhou.

**Supervision:** Li Zhou.

**Validation:** Li Zhou.

**Writing – original draft:** Ailu Wang.

**Writing – review & editing:** Li Zhou.

## Supplementary Material






