# Supplementary material for: Construction of ferroptosis-related prediction model for pathogenesis, diagnosis and treatment of ruptured abdominal aortic aneurysm
Source: Medicine (Baltimore). 2024 May 10;103(19):e38134. doi: 10.1097/MD.0000000000038134 (PMC11081628; doi:10.1097/MD.0000000000038134)
Supplement: Supplementary file 2 [file medi-103-e38134-s002.docx]

**Table S2 21 up-regulated genes and 16 down-regulated genes of DEGs results analyzed between the stable group and the rupture group from GSE98278.**

| **id** | **logFC** | **AveExpr** | **t** | **P.Value** | **adj.P.Val** | **B** |
| --- | --- | --- | --- | --- | --- | --- |
| MT1A | 1.28955 | 12.3808 | 6.50397 | 4.50E-08 | 0.00066 | 7.86075 |
| CX3CR1 | -2.0691 | 5.09632 | -6.4083 | 6.30E-08 | 0.00066 | 7.57421 |
| MPZL2 | 1.34361 | 7.23269 | 6.40781 | 6.31E-08 | 0.00066 | 7.57277 |
| IRAK3 | 1.16395 | 7.65007 | 6.0596 | 2.14E-07 | 0.00168 | 6.52818 |
| MT2A | 1.25961 | 11.8808 | 5.84685 | 4.49E-07 | 0.00257 | 5.88986 |
| CCL8 | -1.628 | 9.08166 | -5.8208 | 4.92E-07 | 0.00257 | 5.8118 |
| MT1X | 1.90789 | 8.51163 | 5.74918 | 6.31E-07 | 0.00283 | 5.59712 |
| CLN6 | -1.8402 | 3.01956 | -5.644 | 9.09E-07 | 0.00324 | 5.28233 |
| ZDHHC9 | 1.04841 | 6.5684 | 5.62447 | 9.73E-07 | 0.00324 | 5.22385 |
| F2RL3 | 2.17346 | 4.21947 | 5.45483 | 1.75E-06 | 0.00499 | 4.71739 |
| OAS3 | -1.4765 | 5.38839 | -5.4276 | 1.92E-06 | 0.00503 | 4.63634 |
| FKBP5 | 1.01086 | 9.00689 | 5.34677 | 2.54E-06 | 0.00583 | 4.39578 |
| S100A4 | -1.011 | 9.45649 | -5.3398 | 2.60E-06 | 0.00583 | 4.3752 |
| IFIT1 | -1.446 | 6.60483 | -5.3087 | 2.89E-06 | 0.00606 | 4.28254 |
| MX1 | -1.0259 | 10.633 | -5.2023 | 4.17E-06 | 0.00818 | 3.96734 |
| MTE | 1.3316 | 8.66893 | 5.1699 | 4.65E-06 | 0.00859 | 3.8716 |
| ANGPTL4 | 1.91015 | 9.79472 | 5.02735 | 7.56E-06 | 0.01319 | 3.45154 |
| SLC39A14 | 1.55894 | 8.45875 | 4.94839 | 9.88E-06 | 0.01633 | 3.21993 |
| NAGA | -2.0453 | 3.99591 | -4.8285 | 1.48E-05 | 0.02112 | 2.86997 |
| DDIT4 | 1.18942 | 10.4438 | 4.78055 | 1.74E-05 | 0.0234 | 2.73067 |
| C7orf68 | 2.01453 | 6.94962 | 4.77198 | 1.79E-05 | 0.0234 | 2.70581 |
| INHBB | 1.40424 | 7.61648 | 4.73068 | 2.05E-05 | 0.0248 | 2.58613 |
| GPR4 | 1.21516 | 6.46833 | 4.61222 | 3.05E-05 | 0.03085 | 2.24462 |
| ADM | 1.24889 | 10.5661 | 4.5878 | 3.30E-05 | 0.0324 | 2.17454 |
| PLEKHA6 | -1.7789 | 4.22428 | -4.5396 | 3.87E-05 | 0.03649 | 2.03664 |
| ZNF618 | -1.1884 | 4.71517 | -4.5334 | 3.95E-05 | 0.03649 | 2.01894 |
| C1orf133 | -1.3336 | 5.53192 | -4.4749 | 4.79E-05 | 0.04066 | 1.85206 |
| RAB7B | -1.6008 | 6.50384 | -4.4512 | 5.18E-05 | 0.04169 | 1.78473 |
| ADAMTS9 | 1.58102 | 8.15528 | 4.42776 | 5.59E-05 | 0.04389 | 1.71826 |
| SYTL3 | 1.41779 | 6.63726 | 4.38673 | 6.40E-05 | 0.0478 | 1.60217 |
| MT1G | 1.43075 | 8.9792 | 4.35946 | 6.99E-05 | 0.04861 | 1.52523 |
| LOC284422 | 1.51382 | 3.44264 | 4.35507 | 7.09E-05 | 0.04861 | 1.51287 |
| NLGN1 | -1.521 | 3.68576 | -4.3429 | 7.38E-05 | 0.04861 | 1.47867 |
| IL1RL1 | 1.82642 | 4.03607 | 4.34218 | 7.39E-05 | 0.04861 | 1.47656 |
| PELI3 | -1.5266 | 3.62212 | -4.3334 | 7.61E-05 | 0.04861 | 1.45179 |
| DDX58 | -1.0286 | 5.21235 | -4.3067 | 8.30E-05 | 0.04883 | 1.37688 |
| PET112L | -1.2414 | 5.14852 | -4.3029 | 8.40E-05 | 0.04883 | 1.36608 |

DEGs= differentially expressed genes.
