# Supplementary material for: Construction of ferroptosis-related prediction model for pathogenesis, diagnosis and treatment of ruptured abdominal aortic aneurysm
Source: Medicine (Baltimore). 2024 May 10;103(19):e38134. doi: 10.1097/MD.0000000000038134 (PMC11081628; doi:10.1097/MD.0000000000038134)
Supplement: Supplementary file 3 [file medi-103-e38134-s003.docx]

**Table S3 Prediction of upstream regulatory miRNAs of MT1G and DDIT4.**

| **MT1G** | **DDIT4** |
| --- | --- |
| hsa-miR-15a-5p | hsa-miR-101-3p.1 |
| hsa-miR-15b-5p | hsa-miR-101-3p.2 |
| hsa-miR-497-5p | hsa-miR-139-5p |
| hsa-miR-424-5p | hsa-miR-221-3p |
| hsa-miR-6838-5p | hsa-miR-222-3p |
| hsa-miR-195-5p | hsa-miR-802 |
| hsa-miR-16-5p | hsa-miR-3129-5p |
| hsa-miR-96-5p | hsa-miR-199a-3p |
| hsa-miR-1271-5p | hsa-miR-199b-3p |
| hsa-miR-183-5p.2 | hsa-miR-22-3p |
| hsa-miR-298 | hsa-miR-181c-5p |
| hsa-miR-519b-3p | hsa-miR-181d-5p |
| hsa-miR-519c-3p | hsa-miR-181a-5p |
| hsa-miR-519a-3p | hsa-miR-181b-5p |
| hsa-miR-20b-5p | hsa-miR-4262 |
| hsa-miR-20a-5p | hsa-miR-183-5p.2 |
| hsa-miR-17-5p | hsa-miR-183-5p.1 |
| hsa-miR-106a-5p | hsa-miR-7-5p |
| hsa-miR-106b-5p | hsa-miR-30e-5p |
| hsa-miR-519d-3p | hsa-miR-30d-5p |
| hsa-miR-526b-3p | hsa-miR-30a-5p |
| hsa-miR-93-5p | hsa-miR-30b-5p |
| hsa-miR-3978 | hsa-miR-30c-5p |
| hsa-miR-875-3p | hsa-miR-429 |
| hsa-miR-4308 | hsa-miR-200b-3p |
| hsa-miR-6779-3p | hsa-miR-200c-3p |
| hsa-miR-1224-3p | hsa-miR-374c-5p |
| hsa-miR-4758-3p | hsa-miR-655-3p |
| hsa-miR-5699-5p | hsa-miR-153-3p |
| hsa-miR-3189-5p | hsa-miR-448 |
| hsa-miR-1913 | hsa-miR-25-3p |
| hsa-miR-324-3p | hsa-miR-367-3p |
| hsa-miR-22-3p | hsa-miR-32-5p |
| hsa-miR-320b | hsa-miR-92b-3p |
| hsa-miR-320a | hsa-miR-92a-3p |
| hsa-miR-320d | hsa-miR-363-3p |
| hsa-miR-320c | hsa-miR-495-3p |
| hsa-miR-4429 | hsa-miR-5688 |
| hsa-miR-539-5p | hsa-miR-6075 |
| hsa-miR-580-3p | hsa-miR-6760-3p |
| hsa-miR-3121-5p | hsa-miR-3616-3p |
| hsa-miR-193a-5p | hsa-miR-6515-5p |
| hsa-miR-4506 | hsa-miR-1249-5p |
| hsa-miR-613 | hsa-miR-6797-5p |
| hsa-miR-206 | hsa-miR-4443 |
| hsa-miR-1-3p | hsa-miR-432-5p |
| hsa-miR-6505-5p | hsa-miR-431-5p |
| hsa-miR-3138 | hsa-miR-3120-5p |
| hsa-miR-8071 | hsa-miR-6888-3p |
| hsa-miR-3165 | hsa-miR-593-3p |
| hsa-miR-6880-5p | hsa-miR-5000-5p |
| hsa-miR-4456 | hsa-miR-4474-5p |
| hsa-miR-582-3p | hsa-miR-554 |
| hsa-miR-549a | hsa-miR-6781-3p |
| hsa-miR-4768-3p | hsa-miR-2682-3p |
| hsa-miR-4433a-3p | hsa-miR-6747-3p |
| hsa-miR-4459 | hsa-miR-6727-3p |
| hsa-miR-3622b-5p | hsa-miR-4722-3p |
| hsa-miR-433-3p | hsa-miR-1976 |
| hsa-miR-7154-5p | hsa-miR-6736-3p |
| hsa-miR-1206 | hsa-miR-6787-3p |
| hsa-miR-5000-5p | hsa-miR-2114-5p |
| hsa-miR-4751 | hsa-miR-1910-5p |
| hsa-miR-6847-5p | hsa-miR-455-3p.1 |
| hsa-miR-4323 | hsa-miR-140-3p.1 |
| hsa-miR-6720-5p | hsa-miR-140-3p.2 |
| hsa-miR-6512-3p | hsa-miR-4328 |
| hsa-miR-6849-3p | hsa-miR-6882-3p |
| hsa-miR-766-3p | hsa-miR-647 |
| hsa-miR-1470 | hsa-miR-604 |
| hsa-miR-4667-3p | hsa-miR-6762-3p |
| hsa-miR-136-5p | hsa-miR-6865-5p |
| hsa-miR-5699-3p | hsa-miR-6815-5p |
| hsa-miR-4421 | hsa-miR-6802-5p |
| hsa-miR-214-5p | hsa-miR-6800-5p |
| hsa-miR-6735-3p | hsa-miR-1306-5p |
| hsa-miR-6793-3p | hsa-miR-1224-3p |
| hsa-miR-5699-5p | hsa-miR-361-3p |
| hsa-miR-3189-5p | hsa-miR-3679-3p |
| hsa-miR-4758-3p | hsa-miR-3938 |
| hsa-miR-1913 | hsa-miR-6731-3p |
| hsa-miR-324-3p | hsa-miR-4261 |
| hsa-miR-6752-3p | hsa-miR-6733-5p |
| hsa-miR-7113-3p | hsa-miR-4668-5p |
| hsa-miR-146b-5p | hsa-miR-3153 |
| hsa-miR-146a-5p | hsa-miR-6739-5p |
| hsa-miR-7153-5p | hsa-miR-4646-5p |
| hsa-miR-3925-5p | hsa-miR-204-3p |
| hsa-miR-3919 | hsa-miR-1343-3p |
| hsa-miR-4756-3p | hsa-miR-6783-3p |
| hsa-miR-383-5p.1 | hsa-miR-6742-3p |
| hsa-miR-4771 | hsa-miR-6852-5p |
| hsa-miR-4468 | hsa-miR-939-3p |
| hsa-miR-2278 | hsa-miR-7162-5p |
| hsa-miR-6501-3p | hsa-miR-516a-3p |
| hsa-miR-6801-5p | hsa-miR-516b-3p |
| hsa-miR-4461 | hsa-miR-27b-5p |
| hsa-miR-5186 | hsa-miR-556-5p |
| hsa-miR-6888-5p | hsa-miR-4691-3p |
| hsa-miR-4428 | hsa-miR-6793-3p |
| hsa-miR-766-5p | hsa-miR-3657 |
| hsa-miR-7106-5p | hsa-miR-4669 |
| hsa-miR-6825-5p | hsa-miR-511-3p |
| hsa-miR-3150a-3p | hsa-miR-5194 |
| hsa-miR-6763-5p | hsa-miR-6738-5p |
| hsa-miR-3175 | hsa-miR-1914-3p |
| hsa-miR-939-5p | hsa-miR-6734-5p |
| hsa-miR-1343-5p | hsa-miR-3184-5p |
| hsa-miR-4667-5p | hsa-miR-423-5p |
| hsa-miR-4700-5p | hsa-miR-1278 |
| hsa-miR-8089 | hsa-miR-144-3p |
| hsa-miR-6852-5p | hsa-miR-548v |
| hsa-miR-661 | hsa-miR-187-5p |
| hsa-miR-6081 | hsa-miR-221-3p |
| hsa-miR-4722-5p | hsa-miR-222-3p |
| hsa-miR-6165 | hsa-miR-7109-3p |
| hsa-miR-3619-5p | hsa-miR-874-5p |
| hsa-miR-214-3p | hsa-miR-328-3p |
| hsa-miR-761 | hsa-miR-4312 |
| hsa-miR-7843-5p | hsa-miR-671-5p |
| hsa-miR-6735-5p | hsa-miR-6079 |
| hsa-miR-6879-5p | hsa-miR-6828-5p |
| hsa-miR-4632-5p | hsa-miR-1207-3p |
| hsa-miR-4436b-3p | hsa-miR-4267 |
| hsa-miR-140-3p.1 | hsa-miR-5586-5p |
| hsa-miR-4690-3p | hsa-miR-8485 |
| hsa-miR-5685 | hsa-miR-8485 |
| hsa-miR-6856-3p | hsa-miR-3941 |
| hsa-miR-3614-5p | hsa-miR-362-3p |
| hsa-miR-216b-3p | hsa-miR-603 |
| hsa-miR-8064 | hsa-miR-329-3p |
| hsa-miR-3187-5p | hsa-miR-187-5p |
| hsa-miR-5189-5p | hsa-miR-548v |
| hsa-miR-612 | hsa-miR-548ag |
| hsa-miR-1285-3p | hsa-miR-570-5p |
| hsa-miR-6860 | hsa-miR-548ai |
| hsa-miR-939-3p | hsa-miR-548ba |
| hsa-miR-6791-5p | hsa-miR-5692a |
| hsa-miR-4292 | hsa-miR-7-2-3p |
| hsa-miR-6779-3p | hsa-miR-5688 |
| hsa-miR-7156-3p | hsa-miR-7-1-3p |
| hsa-miR-1273h-3p | hsa-miR-495-3p |
| hsa-miR-3678-3p | hsa-miR-545-5p |
| hsa-miR-6516-5p | hsa-miR-223-3p |
| hsa-miR-3121-5p | hsa-miR-4697-3p |
| hsa-miR-210-5p | hsa-miR-1208 |
| hsa-miR-4749-3p | hsa-miR-1228-3p |
| hsa-miR-4325 | hsa-miR-4764-5p |
| hsa-miR-4793-3p | hsa-miR-378j |
| hsa-miR-646 | hsa-miR-5706 |
| hsa-miR-3937 | hsa-miR-4782-5p |
| hsa-miR-3913-3p | hsa-miR-6839-5p |
| hsa-miR-4449 | hsa-miR-518c-5p |
| hsa-miR-4675 | hsa-miR-6824-3p |
| hsa-miR-4741 | hsa-miR-3191-5p |
| hsa-miR-4446-3p | hsa-miR-6764-3p |
| hsa-miR-6132 | hsa-miR-330-5p |
| hsa-miR-6836-5p | hsa-miR-326 |
| hsa-miR-6510-5p | hsa-miR-6817-3p |
| hsa-miR-6081 | hsa-miR-4690-3p |
| hsa-miR-6090 | hsa-miR-5685 |
| hsa-miR-6726-5p | hsa-miR-6081 |
| hsa-miR-5591-5p | hsa-miR-3196 |
| hsa-miR-920 | hsa-miR-3180 |
| hsa-miR-4300 | hsa-miR-6816-5p |
| hsa-miR-6827-5p | hsa-miR-3180-3p |
| hsa-miR-30c-2-3p | hsa-miR-4731-5p |
| hsa-miR-30c-1-3p | hsa-miR-4505 |
| hsa-miR-6788-5p | hsa-miR-5787 |
| hsa-miR-887-5p | hsa-miR-4498 |
| hsa-miR-1273h-5p | hsa-miR-5001-5p |
| hsa-miR-6779-5p | hsa-miR-4492 |
| hsa-miR-30b-3p | hsa-miR-762 |
| hsa-miR-3689a-3p | hsa-miR-6781-5p |
| hsa-miR-3689b-3p | hsa-miR-6877-5p |
| hsa-miR-3689c | hsa-miR-939-3p |
| hsa-miR-6780a-5p | hsa-miR-6791-5p |
| hsa-miR-890 | hsa-miR-4292 |
| hsa-miR-138-2-3p | hsa-miR-504-5p.1 |
| hsa-miR-6794-3p | hsa-miR-3620-3p |
| hsa-miR-4639-3p | hsa-miR-6865-3p |
| hsa-miR-668-3p | hsa-miR-6802-3p |
| hsa-miR-1290 | hsa-miR-6754-3p |
| hsa-miR-3167 | hsa-miR-412-3p |
| hsa-miR-876-5p | hsa-miR-205-5p |
| hsa-miR-378j | hsa-miR-6868-3p |
| hsa-miR-6839-5p | hsa-miR-1236-3p |
| hsa-miR-4254 | hsa-miR-877-3p |
| hsa-miR-3690 | hsa-miR-2682-3p |
| hsa-miR-4668-3p | hsa-miR-6781-3p |
| hsa-miR-548c-3p | hsa-miR-6767-3p |
| hsa-miR-3613-3p | hsa-miR-4689 |
| hsa-miR-3613-3p | hsa-miR-3150b-3p |
| hsa-miR-548p | hsa-miR-6858-5p |
|  | hsa-miR-4784 |
|  | hsa-miR-7162-3p |
|  | hsa-miR-4649-3p |
|  | hsa-miR-3921 |
|  | hsa-miR-4653-5p |
|  | hsa-miR-3194-3p |
|  | hsa-miR-6805-3p |
|  | hsa-miR-5691 |
|  | hsa-miR-6762-3p |
|  | hsa-miR-1470 |
|  | hsa-miR-4667-3p |
|  | hsa-miR-4469 |
|  | hsa-miR-6887-3p |
|  | hsa-miR-1306-5p |
|  | hsa-miR-6776-3p |
|  | hsa-miR-497-3p |
|  | hsa-miR-6715a-3p |
|  | hsa-miR-4731-3p |
|  | hsa-miR-4801 |
|  | hsa-miR-4662a-3p |
|  | hsa-miR-5088-3p |
|  | hsa-miR-133a-3p.1 |
|  | hsa-miR-4281 |
|  | hsa-miR-133a-3p.2 |
|  | hsa-miR-133b |
|  | hsa-miR-3614-5p |
|  | hsa-miR-216b-3p |
|  | hsa-miR-603 |
|  | hsa-miR-495-3p |
|  | hsa-miR-5688 |
|  | hsa-miR-3065-5p |
|  | hsa-miR-3529-3p |
|  | hsa-miR-495-3p |
|  | hsa-miR-5688 |
|  | hsa-miR-2115-3p |
|  | hsa-miR-383-5p.1 |
|  | hsa-miR-8062 |
|  | hsa-miR-8079 |
|  | hsa-miR-1245a |
|  | hsa-miR-6073 |
|  | hsa-miR-936 |
|  | hsa-miR-222-5p |
|  | hsa-miR-3529-3p |
|  | hsa-miR-24-1-5p |
|  | hsa-miR-24-2-5p |
|  | hsa-miR-6882-3p |
|  | hsa-miR-4662a-3p |
|  | hsa-miR-3607-3p |
|  | hsa-miR-3686 |
|  | hsa-miR-600 |
|  | hsa-miR-3685 |
|  | hsa-miR-155-3p |
|  | hsa-miR-3667-3p |
|  | hsa-miR-6756-3p |
|  | hsa-miR-3127-3p |
|  | hsa-miR-6730-3p |
|  | hsa-miR-4732-3p |
|  | hsa-miR-345-3p |
|  | hsa-miR-670-5p |
|  | hsa-miR-4319 |
|  | hsa-miR-125b-5p |
|  | hsa-miR-125a-5p |
|  | hsa-miR-8079 |
|  | hsa-miR-1245a |
|  | hsa-miR-6814-5p |
|  | hsa-miR-4733-5p |
|  | hsa-miR-6513-3p |
|  | hsa-miR-26a-5p |
|  | hsa-miR-4465 |
|  | hsa-miR-26b-5p |
|  | hsa-miR-1297 |
|  | hsa-miR-498 |
|  | hsa-miR-1305 |
|  | hsa-miR-335-3p |
|  | hsa-miR-4775 |
|  | hsa-miR-590-3p |
|  | hsa-miR-3934-5p |
|  | hsa-miR-5006-5p |
|  | hsa-miR-4755-3p |
|  | hsa-miR-22-3p |
|  | hsa-miR-302a-5p |
|  | hsa-miR-4747-5p |
|  | hsa-miR-5196-5p |
|  | hsa-miR-6780b-5p |
|  | hsa-miR-4725-3p |
|  | hsa-miR-4271 |
|  | hsa-miR-625-5p |
|  | hsa-miR-1275 |
|  | hsa-miR-4665-5p |
|  | hsa-miR-5010-5p |
|  | hsa-miR-4525 |
|  | hsa-miR-7111-5p |
|  | hsa-miR-4723-5p |
|  | hsa-miR-5698 |
|  | hsa-miR-6870-5p |
|  | hsa-miR-17-3p |
|  | hsa-miR-3158-5p |
|  | hsa-miR-1184 |
|  | hsa-miR-1205 |
|  | hsa-miR-4267 |
|  | hsa-miR-1202 |
|  | hsa-miR-3972 |
|  | hsa-miR-3194-5p |
|  | hsa-miR-6727-3p |
|  | hsa-miR-4722-3p |
|  | hsa-miR-6752-3p |
|  | hsa-miR-6801-3p |
|  | hsa-miR-6810-3p |
|  | hsa-miR-331-3p |
|  | hsa-miR-6887-3p |
|  | hsa-miR-4749-3p |
|  | hsa-miR-6769a-3p |
|  | hsa-miR-4690-3p |
|  | hsa-miR-5685 |
|  | hsa-miR-6787-3p |
|  | hsa-miR-6892-3p |
|  | hsa-miR-6865-3p |
|  | hsa-miR-3943 |
|  | hsa-miR-6512-3p |
|  | hsa-miR-6720-5p |
|  | hsa-miR-6849-3p |
|  | hsa-miR-23b-5p |
|  | hsa-miR-23a-5p |
|  | hsa-miR-6738-5p |
|  | hsa-miR-5194 |
|  | hsa-miR-1914-3p |
|  | hsa-miR-4728-5p |
|  | hsa-miR-6785-5p |
|  | hsa-miR-6883-5p |
|  | hsa-miR-149-3p |
|  | hsa-miR-6825-5p |
|  | hsa-miR-6889-5p |
|  | hsa-miR-6777-5p |
|  | hsa-miR-4532 |
|  | hsa-miR-1247-3p |
|  | hsa-miR-6749-3p |
|  | hsa-miR-483-3p.1 |
|  | hsa-miR-483-3p.2 |
|  | hsa-miR-1912 |
|  | hsa-miR-1295b-5p |
|  | hsa-miR-3667-5p |
|  | hsa-miR-6077 |
|  | hsa-miR-4777-5p |
|  | hsa-miR-523-5p |
|  | hsa-miR-522-5p |
|  | hsa-miR-519b-5p |
|  | hsa-miR-519c-5p |
|  | hsa-miR-518e-5p |
|  | hsa-miR-519a-5p |
|  | hsa-miR-518d-5p |
|  | hsa-miR-518f-5p |
|  | hsa-miR-526a |
|  | hsa-miR-520c-5p |
|  | hsa-miR-3168 |
|  | hsa-miR-4277 |
|  | hsa-miR-4495 |
|  | hsa-miR-6516-3p |
|  | hsa-miR-141-5p |
|  | hsa-miR-200b-5p |
|  | hsa-miR-200a-5p |
|  | hsa-miR-496.1 |
|  | hsa-miR-3174 |
|  | hsa-miR-7154-5p |
|  | hsa-miR-8060 |
|  | hsa-miR-433-3p |
|  | hsa-miR-664a-3p |
|  | hsa-miR-630 |
|  | hsa-miR-223-5p |
|  | hsa-miR-6513-3p |
|  | hsa-miR-8084 |
|  | hsa-miR-559 |
|  | hsa-miR-548au-5p |
|  | hsa-miR-548ae-5p |
|  | hsa-miR-548y |
|  | hsa-miR-548ak |
|  | hsa-miR-548j-5p |
|  | hsa-miR-548a-5p |
|  | hsa-miR-548ap-5p |
|  | hsa-miR-548am-5p |
|  | hsa-miR-548h-5p |
|  | hsa-miR-548i |
|  | hsa-miR-548ab |
|  | hsa-miR-548b-5p |
|  | hsa-miR-548as-5p |
|  | hsa-miR-548o-5p |
|  | hsa-miR-548ad-5p |
|  | hsa-miR-548w |
|  | hsa-miR-548ay-5p |
|  | hsa-miR-548c-5p |
|  | hsa-miR-548aq-5p |
|  | hsa-miR-548bb-5p |
|  | hsa-miR-548d-5p |
|  | hsa-miR-548ar-5p |
|  | hsa-miR-548n |
|  | hsa-miR-7-1-3p |
|  | hsa-miR-7-2-3p |
|  | hsa-miR-302c-5p |
|  | hsa-miR-552-5p |
|  | hsa-miR-452-5p |
|  | hsa-miR-892c-3p |
|  | hsa-miR-4676-3p |
